# Crisis Preparedness of Healthcare Manufacturing Firms during the COVID-19 Outbreak: Digitalization and Servitization

**DOI:** 10.3390/ijerph18105456

**Published:** 2021-05-20

**Authors:** Jingsi Zhang, Liangqun Qi

**Affiliations:** School of Economics and Management, Harbin University of Science and Technology, Harbin 150080, China; jingsi199@163.com

**Keywords:** healthcare manufacturing firms, organizational resilience, COVID-19, servitization, digitalization

## Abstract

For healthcare manufacturing firms, creating a crisis-prepared product and service portfolio and operational processes is essential for their long-term prosperity. In this paper, we examine how healthcare manufacturing firms cope with the operational disruptions and opportunities associated with the COVID-19 pandemic. We highlight the central role of organizational resilience and examine whether servitization and digitalization can improve the organizational resilience of healthcare manufacturing firms. On the basis of the organizational information processing theory, we suggest that servitization and digitalization can improve the stability and flexibility of operations, which make healthcare manufacturing firms more resilient to the COVID-19 pandemic. The hypotheses were tested using survey data from 163 manufacturing firms located in China. The results indicate that both servitization and digitalization improve the organizational resilience of healthcare manufacturing firms, leading to higher firm growth during the COVID-19 pandemic. Moreover, organizational resilience mediates the impacts of servitization and digitalization on firm growth. Environmental dynamism strengthens the relationship between digitalization and organizational resilience. This study offers new insights for healthcare manufacturing firms to prepare for crisis events and achieve sustainable development in a highly competitive environment.

## 1. Introduction

The outbreak of COVID-19 has caused widespread disruptions in global economic activities. Due to disruptions for upstream suppliers and a decrease in customer demand caused by lockdowns, many healthcare manufacturing firms have had to cease production, and some have even been bankrupted during the outbreak [1,2]. However, some firms have identified opportunities arising from this global health crisis and have improved their competitive advantage. For example, the market value of Samsung Biologics, a subsidiary of Samsung that focuses on manufacturing biopharmaceuticals on a contract basis, increased by USD 20 billion in 2020. Similarly, Dexcom, a leading blood sugar monitor manufacturer, doubled its market value during 2020 [3]. In view of the differing outcomes for firms that face the same crisis, mitigating the negative impact of the COVID-19 outbreak and identifying opportunities from this crisis to achieve sustainable development have become core issues for healthcare manufacturing firms. 

To cope with adversities, previous studies have emphasized the importance of organizational resilience, defined as the ability of an organization to maintain functions and to recover rapidly from adversity by mobilizing and accessing the resources needed [4]. For healthcare manufacturing firms, resilience has played a significant role in achieving sustainable development during the COVID-19 pandemic because this health crisis has also provided opportunities for them to build their competitive advantages [2,5]. According to Williams et al. [6], organizational resilience is context-based. Whereas one firm may be resilient to one type of adversity, it might be less resilient to other types. Therefore, strategies that make firms more resilient to normal disruptions might not necessarily improve organizational resilience to the COVID-19 outbreak, which has caused unprecedented disruption and has had a long-term impact on the manners in which people live and firms operate [7]. Without a comprehensive understanding of its antecedents, firms are unclear about how to build organizational resilience to reduce the disruptions caused by the COVID-19 pandemic. 

Among the top 100 companies that have prospered during the COVID-19 pandemic [3], the majority have built their businesses on services and invested significant resources in digital technologies before the pandemic. This highlights the potential benefits of servitization and digitalization for dealing with the disruptions caused by the COVID-19 pandemic. Servitization is defined as the process of building revenue streams for manufacturing firms from services [8]. It increases the sources of revenue and can overcome the disruption of one market segment. Digitalization refers to the use of digital technologies to innovate a business model and to provide new revenue streams and value-producing opportunities in industrial ecosystems [9]. The adoption of digital technologies improves the information processing capability of firms and enables the efficient use of resources [10,11]. From this perspective, servitization and digitalization provide benefits for recovering from adversities. However, from the resource dependence perspective [12], servitization and digitalization increase the dependence on supply chain partners and hinder the flexibility to cope with exogenous shocks, such as the COVID-19 outbreak. For example, firms with a service-based business model tend to integrate closely with customers, and thus are more prone to disruptions of market demand [13]. Similarly, digitalization might increase infrastructural investments that prevent firms from making quick changes to environmental shocks [9]. The mixed evidence about the benefits and disadvantages of servitization and digitalization calls for more empirical studies on testing how servitization and digitalization affect the organizational resilience of manufacturing firms. Such empirical evidence would help manufacturing firms to make evidence-based decisions about servitization and digitalization. 

In view of the importance of service and digital technologies for firms to recover from the disruptions caused by the pandemic [14], this study examines how servitization and digitalization affect the organizational resilience and firm growth of healthcare manufacturing firms during the COVID-19 pandemic. According to organizational information processing theory (OIPT), we suggest that servitization and digitalization improved the organizational resilience of healthcare manufacturing firms, leading to higher firm growth during the COVID-19 pandemic. Specifically, servitization facilitates the diversification of business models and reduces the risk of relying on one business segment, leading to the improvement of operational process stability [14]. Digitalization improves the information-processing capability, which helps firms to anticipate, make rapid decisions, and adjust proactively to reduce the negative impacts of adverse events [15]. Furthermore, we also suggest that diversification of business models and information-processing capabilities is essential when firms operate in dynamic environments. The hypotheses were tested based on a survey of 163 Chinese medical device manufacturing firms. 

This study contributes to the existing literature in three ways. First, it addresses the need for more studies on coping with the disruptions caused by the COVID-19 pandemic [1]. Unlike other short-term environmental jolts [16], the COVID-19 pandemic has had a global and long-term impact on the way firms operate. As a result, strategies for dealing with other environmental effects do not necessarily apply to the COVID-19 pandemic. This study indicates that servitization and digitalization improved organizational resilience, which helped manufacturing firms to achieve higher growth during the COVID-19 pandemic. 

Second, extending previous studies on the antecedents of organizational resilience [17,18], this study finds that servitization and digitalization acted as key determinants of organizational resilience during the COVID-19 pandemic. Consistent with previous studies that emphasized information processing capabilities in uncertain environments [15,19], we also found that the effect of digitalization was more prominent for firms that operated in industries characterized by high environmental dynamism. 

Third, this study offers a new perspective for understanding the performance implications of servitization and digitalization. From the organizational resilience perspective, this study introduces organizational resilience as an intermediary variable to explain the mechanisms through which servitization and digitalization improve firm growth during the COVID-19. The findings extend previous studies that have focused on the direct impacts of servitization and digitalization on performance indicators [20]. 

This article is organized as follows: In Section 2, we review previous studies on organizational resilience, servitization, and digitalization and based on the literature review, we propose hypotheses in Section 3; in Section 4, we present data collection and measurement of variables; in Section 5, we present our results; in Section 6, we discuss the theoretical and practical implications of the results; finally, we provide conclusions, limitations, and future research suggestions. 

## 2. Literature Review

### 2.1. Organizational Resilience

The notion of resilience was proposed by ecology researchers who attempted to examine how ecological systems responded to external disturbances. The concept was later introduced in other areas such as engineering, economics, psychology, management, and socioecology [6]. Management studies on resilience can be divided into those studies at the individual level [21] and those at the organizational level of resilience [4]. The individual-level studies of resilience are rooted in positive psychology and focus on how employee resilience contributes to organizational commitment and job satisfaction. At the individual level, resilience refers to organizational members’ ability to bounce back, or even succeed, in the face of adversity [21]. In contrast, organizational-level studies focus on an organization’s ability to respond more quickly and recover faster from unseen disruption [22]. In this study, we focus on the growing body of work on organizational resilience. 

Previous management studies have viewed resilience from two different perspectives, i.e., the static and the dynamic viewpoints [23]. In the static viewpoint, resilience represents a firm’s ability to recover from disruptions and is limited to the phase of coping with adverse events [11]. In contrast, the dynamic view of resilience has a broader focus and views resilience as the ability to cope with threats and opportunities in external environments. This perspective has a broader view that covers coping with adverse events and also how organizations prepare for unknown adverse events and learn from them [24]. In this article, we focus on the interaction between the firm and the environment and adopt the dynamic view of organizational resilience. Consistent with Hillmann and Guenther [4], we define organizational resilience as the ability of an organization to maintain functions and recover rapidly from adversity by mobilizing and accessing the resources needed. 

Although resilience has been conceptualized in different ways in previous studies, it contained the core element of stability and flexibility [17]. Stability refers to a firm’s ability to maintain a stable flow of its processes and maintain its organizational attributes. Flexibility refers to a firm’s ability to innovate and adapt to changes. According to Jüttner and Maklan [25], four organizational capabilities, i.e., flexibility, velocity/reaction speed, access to timely information, and collaborations among supply chain members, are critical for firms to minimize the impact of adverse events. Proactive risk management and resource reconfiguration are also important for a firm to cope with operational disruptions [18]. Although the findings of previous studies on organizational resilience have been consistent in terms of the outcomes of organizational resilience, factors that improve organizational resilience remain to be inconclusive. This inconsistency is more prominent in the context of medical device manufacturing firms facing the COVID-19 outbreak, which has both threatened short-term survival and provided opportunities for the long-term prosperity of the healthcare industry. Therefore, more research is needed to examine the antecedents and boundary conditions of organizational resilience of medical device manufacturing firms during the COVID-19 outbreak. 

### 2.2. Organizational Information Processing Theory and Organizational Resilience

The OIPT focuses on the role of information that helps firms to mitigate environmental uncertainties [26]. Firms that operate in uncertain environments can either reduce their needs for information through a “mechanistic” organizational structure, or increase information processing capabilities to organize and use information effectively [27]. The OIPT provides a promising perspective for understanding the critical role of information in organizational decision making. From the OIPT, firms operating in dynamic and competitive business environments should constantly collect, analyze, and use information effectively to mitigate environment uncertainty. Firms with a strong information processing capability tend to be more efficient and can achieve a high level of firm performance [26]. The OIPT has been used to understand the performance implications of a wide variety of operational practices and capabilities, such as supply chain resilience [28], analytics capability [26], and big data analytics capability [29]. For example, based on the OIPT, big data analytics capability can improve hospital-patient integration, inter-functional integration, and hospital-supplier integration of the hospital supply chain, leading to the improvement of hospital operational efficiency [30]. From the OIPT, organizational resilience can be built based on a firm’s information processing capability [28]. Firms with strong information processing capability, which enables them to effectively collect, analyze, and use information, are more capable of predicting environmental changes and make proactive adjustments to adapt to environmental changes [29]. 

### 2.3. Servitization and Digitalization 

Servitization refers to the process of building revenue streams for manufacturing firms from services [8]. To cope with the increasingly diversified customer needs and intensive competition in the product sector, many manufacturing firms have transformed their product-based business models to a combination of product and service business models to create sustainable competitive advantages. Servitization has gained popularity in recent years as manufacturing firms have increasingly diversified their business models to increase profit margins, gain loyal customers, and build stable cash flows. Servitization can also be regarded as a service-based business model innovation [31]. During the servitization process, manufacturing firms create value by adding services to their product portfolios. This can be considered to be a change in the way firms create, deliver, and capture value [32]. During this business model innovation process, manufacturing firms create value to customers by adding services to their product portfolios [33]. For example, in addition to selling printers to customers, Xerox also offers printer renting and material supply and maintenance services to its customers [34]. Similarly, in the aircraft engine industry, the “power-by-the-hour” business model has been widely used by engine manufacturers, such as GM and Rolls Royce. In the “power-by-the-hour” business model, airline companies no longer “own” the engines. Instead, they pay according to the hours their aircraft have been powered by the engine [35]. Previous studies have shown that servitization can bring market, financial, and strategic benefits to manufacturing firms. First, adding services to products can improve the performance of products and reduce customer complaints when products fail. As a result, adding services to products can improve customer loyalty, which, in turn, helps firms to increase sales because loyal customers are more likely to purchase goods and services from the same company [36]. Second, as compared with product-based business models, service-based business models tend to have a higher profit margin [37]. Third, servitization can help firms to build sustainable competitive advantages because service-based business models are less imitable than business models based on products. According to the resource-based view, resources that are valuable, rare, imperfectly imitable, and not substitutable can help firms to build sustainable competitive advantages [38]. Consistent with this perspective, the resources associated with services are labor intensive and tend to be embedded in employee skills and implicit knowledge. As a result, resources related to services are less imitable than resources associated with products [39]. Therefore, servitization can help firms to increase the competitive barrier and competitive advantages based on services that are more sustainable than those based on products. Consistent with these benefits, empirical research has shown that servitization is positively related to firm performance indicators, such as sales [39] and profitability [37].

Recent studies have emphasized the technological infrastructure for servitization and suggested that digital technologies were integral parts of servitization [13]. To cope with servitization, manufacturing firms have increasingly implemented digitalization to deliver services and to create more value for customers [40,41]. Digitalization is the transformation of business models as a result of a fundamental change to core internal procedures by adopting information and communication technologies [42]. The convergence of the so-called SMAC technologies, i.e., social, mobile, analytics, and cloud computing, has accelerated the process of digitalization. In recent years, digital technologies have been embedded in many aspects of business processes and have become the priorities of many manufacturing firms. Widely used digital technologies, including the Internet of Things (IoT), big data analytics, artificial intelligence (AI), and cloud computing, have changed the way products and services are designed and also the way they are delivered to customers. Digitalization can enable firms to design and deliver novel product service offerings that can create value for customers. For example, IoT has been regarded as foundational to any type of servitization [43]. The adoption of digital technologies can also transform the structure of the supply chain to facilitate the implementation of servitization [10]. As a result, manufacturing firms with the support of digital technologies, such as IoT, cloud computing, and predictive analytics, tend to achieve higher competitiveness in servitization [44].

**Figure 1:**
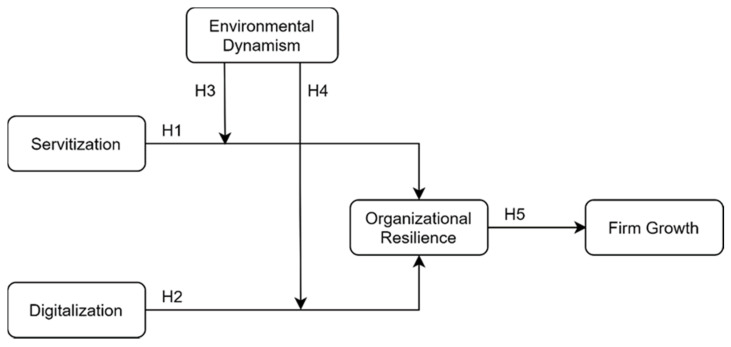
Research model.

Although servitization and digitalization have attracted widespread attention from researchers, previous studies have mainly focused on their instant implications, such as their direct impact on financial indicators [20], whereas their impacts on organizational resilience remain unclear. In this study, we bridge the servitization, digitization, and organizational resilience literature to examine how servitization and digitalization lead to an increase in organizational resilience of manufacturing firms. The research model is presented in Figure 1.

## 3. Hypotheses

### 3.1. Servitization and Organizational Resilience

As compared with traditional manufacturing firms that compete based on product-based business models, servitized manufacturing firms compete based on service-based business models. A comparison of the customer interaction pattern, predominant contractual relationship, operational focus, and competitive positioning is presented in Table 1. In product-based business models, customer interactions are achieved by selling products and negotiating contracts. As a result, the predominant contractual relationship is transactional. Firms focus on assisting the customer in realizing the value potential embedded in products. The value proposition is enhancing the core competitiveness of products [45]. In contrast, in service-based business models, there are higher levels of customer interaction during service delivery and more customer touchpoints [46]. Firms tend to build relational and long-term strategic partnerships with customers [47]. Furthermore, the operational focuses of service-based business models are the co-creation of value in the customer-specific context. Services are differentiated and part of a deliberate service strategy [48]. 

We suggest that manufacturing firms with service-based business models are more resilient to external disruptions than pure product-based manufacturing firms. First, from the OIPT, servitization can broaden the information sources and can enable firms to anticipate environmental changes more effectively. Servitization can facilitate information sharing with supply chain partners, help firms to anticipate environmental change, and to make proactive adjustments to cope with changes. Relational resources are important components of organizational resilience [23]. A key feature of service-based business models is the frequent interaction with customers [49], which enables manufacturing firms to obtain accurate information about the change of customer needs. The customer information gained through servitization helps firms to anticipate market changes and to adjust their operations accordingly to reduce the impacts of these changes. Furthermore, servitization motivates manufacturing firms to enhance stakeholder engagement, which helps them to develop trust with external stakeholders, such as suppliers and customers [50]. Trust helps firms to gain critical information and resources from supply chain partners because partners are more likely to share critical information and key resources with firms they trust [51]. As a result, firms with service-based business models are in a better position to cope with internal and external disruptions. 

Second, firms with diversified business models are in a better position to cope with changes. Firms with more diversified business models, for example, a combination of service-based and product-based business models, have more sources of revenue. Even if one or more of their sources of revenue are negatively affected by negative events, they can gain revenues from other sources [52,53]. In contrast, firms with less diversified business models tend to depend on limited business lines for revenue. After the occurrence of negative events, firms with a small number of revenue sources tend to be more likely to be disrupted [4]. Consistent with this diversification premise, firms with service-based business models tend to have more balanced sources of revenue and can support the operation of manufacturing firms during disruptions from external environments. 

Overall, manufacturing firms with diversified business models can obtain access to more accurate market information that helps them to anticipate and cope with internal and external disruptions. As a result, they tend to have a higher level of organizational resilience than firms with product-based business models. Stated formally:

**Hypothesis** **1** **(H1).** *Servitization is positively related to organizational resilience.*

### 3.2. Digitalization and Organizational Resilience

Digitalization is important for the improvement of organizational resilience. First, from the OIPT, digitalization enhances the information processing capability of a company and helps them to reduce environmental uncertainty. The implementation of digitalization increases a firm’s ability to collect information, and also their ability to process information [11,54]. For example, the implementation of the Internet of Things (IoT) helps firms to gather all types of information, such as that relating to customer needs and product operation conditions, to help firms anticipate the occurrence of internal and external disruptions [43]. Similarly, the adoption of big data analytics enables firms to process massive quantities of data from different sources and helps firms to make optimal decisions to minimize the impact of adverse events [55]. Therefore, firms that implement digitalization tend to be more resilient to adverse events. Second, digitalization enables prompt decision-making, which helps firms to quickly respond to market changes. Efficient decision making has been considered to be the key to reducing the impact of negative events [56]. The implementation of digitalization can help decision-makers to aggregate information and find causal relationships between decision variables and outcomes that help firms to make timely decisions to cope with threats and capture opportunities. Third, digitalization facilitates information sharing within the supply chain and increases supply chain visibility [13]. External collaboration is a core part of organizational resilience. The supply chain integration literature has demonstrated that the adoption of digital technologies, such as Electronic Data Interchange (EDI), enables supply chain partners to share information and help firms to make rapid decisions to avoid the “bullwhip effect” that magnifies the costs of the whole supply chain [57]. Consistent with this rationale, firms that adopt digitalization are more capable of identifying potential disruptions. 

In summary, digitalization increases the information processing capability of manufacturing firms and enables them to improve their decision quality, which further improves their ability to cope with adverse events. Stated formally:

**Hypothesis** **2** **(H2).** *Digitalization is positively related to organizational resilience.*

### 3.3. The Moderating Effect of Environmental Dynamism 

Environmental dynamism represents the magnitude and frequency of environmental change, as well as the irregularity in patterns of environmental change [58]. In a dynamic environment, firms are exposed to frequent changes in customer demand, product and service technologies, and regulations [58]. Moreover, due to frequent environmental changes, competitive advantages are less sustainable in a dynamic environment. From the OIPT, environmental dynamism improves uncertainty and requires a higher level of information processing capability to anticipate changes in environmental environments. Dynamic environments require firms to extensively monitor the changes and collaborate with supply chain partners to obtain more accurate information to assist decision making [59]. In contrast, firms operating in a stable environment face fewer changes and fewer requirements for information processing. 

In a dynamic environment, the diversification of business models is more important for firms to cope with adversities. First, service-based business firms can enable firms to cope with the disruptions caused by environmental changes and to maintain a stable process. The frequent changes in technology and customer demand might threaten the revenue of one stream of a business. As compared with firms with pure product-based models, firms with both service and product revenues can better cope with the environmental changes [14]. In contrast, servitization is less important in a stable environment. In a stable environment, firms are less likely to be disrupted by environmental changes. On the contrary, the resources and investments in servitization might increase opportunity costs and reduce slack resources that can help firms to maintain a stable flow of the process [53]. Second, firms with service-based business models tend to have more information sources to mitigate the uncertainty in a dynamic environment and help firms to build flexibility to cope with changes. The key challenge of a dynamic environment is the unpredictability of environmental change [58]. Firms with service-based business models tend to build strong relationships with suppliers and customers. As a result, they can obtain more accurate information from supply chain partners, and can leverage external resources to mitigate the disruptions and capture the opportunities in a dynamic environment [50]. 

Overall, servitization enhances the stability of operating processes and also improves the flexibility of firms to cope with dynamic environments. Therefore, we suggest that servitization has a stronger impact on organizational resilience in dynamic environments than stable environments. Stated formally:

**Hypothesis** **3** **(H3).** *Environmental dynamism positively moderates the relationship between servitization and organizational resilience such that the positive relationship between servitization and organizational resilience is stronger under high levels of environmental dynamism.*

Firms that operate in dynamic environments require a higher information processing capability. First, the effect of digitalization on proactive anticipation capability is stronger in a dynamic environment. In a dynamic environment, there is a greater level of uncertainty, and mitigation of this uncertainty requires firms to have a stronger information processing capability [60]. Firms equipped with digital technologies can synthesize the information gained from different sources to reduce uncertainties. For example, based on the dynamic capability view of firms, Dubey et al. [61] suggested that digital technologies could enhance the dynamic capability of firms, which was more important for firms that operate in highly dynamic environments. Second, in a dynamic environment, digital technologies are more important for firms to develop the flexibility to adapt to changes. The adoption of digital technologies can facilitate information exchange with external parties. As a result, firms with high levels of digitalization tend to have a stronger absorptive capacity, which enables firms to absorb external knowledge to change their operational processes to cope with the changes. Consistent with this idea, Paiola and Gebauer [43] found that firms equipped with digital technologies such as the Internet of Things (IoT) were more capable of adapting their strategies and business models to cope with rapid technological developments. Similarly, environmental dynamism enhanced the impact of big data analytics and artificial intelligence technologies on operational performance [61]. Overall, digitalization becomes more important for firms to proactively anticipate and reactively respond to adversities. Stated formally:

**Hypothesis** **4** **(H4).** *Environmental dynamism positively moderates the relationship between digitalization and organizational resilience such that the positive relationship between digitalization and organizational resilience is stronger under high levels of environmental dynamism.*

### 3.4. Organizational Resilience and Firm Growth 

During environmental adversities, firms with a higher level of organizational resilience tend to have higher growth. First, organizational resilience has a stabilizing effect that enables firms to operate in a stable flow and avoid operational inefficiencies caused by disruptions [17]. A stable flow of operations has been regarded as the key to achieving high operational efficiency and long-term sustainable growth. Firms with higher levels of organizational resilience are associated with less financial volatility, a higher growth rate, and a higher survival rate in the long term than firms with low levels of organizational resilience [62]. Firms with a high level of organizational resilience tend to be more resistant to adversities and are more capable of adapting to environmental changes. During the global financial crisis in 2008, firms with high levels of organizational resilience experienced smaller losses in value and also took less time to recover from the crisis [17]. As a result, firms with high levels of organizational resilience can maintain a stable operation, which enables them to offset the operational inefficiencies caused by disruptions. 

Second, firms with high levels of organizational resilience are better able to capture opportunities from market changes. Environmental changes create both threats and opportunities for firms [16]. For example, after the outbreak of COVID-19, many cities introduced lockdown restrictions to reduce the spread of coronavirus. These restrictions disrupted the retail industry. However, the pandemic also created opportunities for online retail. During the COVID-19 pandemic, retail companies with online businesses and the innovation capability to build online platforms were more resilient to the pandemic and better able to capture the opportunities for online retailing. 

Finally, resources that help to build organizational resilience are antecedents of organizational creativity, which is the key to high firm growth. For example, relational resources help firms to leverage partners’ capabilities to develop plans to cope with disruptions [63]. Furthermore, information and knowledge from partners are key elements of organizational creativity. As a result, firms with higher levels of organizational resilience tend to have high levels of organizational creativity and are more capable of creating innovative approaches to cope with market changes [23]. Firms with higher levels of organizational creativity tend to have higher growth rates in the long term. 

Overall, firms with high levels of organizational resilience can reduce losses caused by adverse events and capture growth opportunities from environmental changes. Therefore, firms with high levels of organizational resilience tend to have higher growth rates. Stated formally:

**Hypothesis** **5** **(H5).** *Organizational resilience is positively related to firm growth.*

## 4. Methodology

### 4.1. Context and Data Collection

We tested the hypotheses based on data collected through a survey of medical device manufacturing firms. Surveys are advantageous in measuring operational practices adopted by firms as this information is difficult to acquire from secondary sources [64]. In some scenarios, measuring constructs using objective data acquired from secondary sources might introduce measurement errors. In the context of this study, surveys can capture the nuances of servitization and digitalization. 

We acquired the initial list of medical device manufacturing firms with help from governmental institutions. Then, we contacted these firms to see if they were interested in participating in the survey, after which we distributed 286 surveys via express delivery. We also included a postage-paid envelope to help respondents to return the questionnaires. In total, 163 questionnaires with valid information were returned. The product categories of the sample are presented in Table 2. The firm size distribution (based on the number of employees) of the sample is presented in Table 3.

The items in our questionnaires were originally written in English. We translated the questionnaire into Chinese during data collection. Following common practices in survey studies, we adopted a two-step procedure to ensure the proper translation of survey items. First, we asked a translator to translate the English questionnaires into a Chinese questionnaire. Then, we asked another English professional to translate the Chinese questionnaires into English. No significant difference was found despite the choice of words. 

To identify non-response bias, we compared two objective measures, i.e., firm size (measured by the number of employees) and profit margin (measured by return on sales). The results indicated that there were not any significant differences between early and late respondents in terms of firm size and profit margin. We also compared the registered capital and the number of patents of respondent firms and non-respondent firms. The results indicated that respondent firms and non-respondent firms were not significantly different in terms of registered capital and the number of patents. The results indicated that a non-response bias was not a serious concern in this study. 

### 4.2. Measurements

#### 4.2.1. Firm Growth

Consistent with Chen et al. [55], this study measured firm growth from three aspects, namely, sales growth, market expansion, and market share growth. To account for product segment differences in terms of the degree of impact caused by COVID-19, we asked respondents to compare these aspects with other firms within the industry (where 1 = in the bottom 20%, 3 = in the middle 20%, and 5 = in the top 20% of industry performers). To mitigate concerns regarding common method bias, we collected objective firm growth data (sales growth compared to 2019) and compared it with our measure. The result shows that our measure was positively and significantly related to the objective measure of firm growth. 

#### 4.2.2. Organizational Resilience

This study measured organizational resilience based on the scales developed by Parker and Ameen [18]. Specifically, organizational resilience was measured based on the degree to which firms could deal with the business changes, adapt business operations to the disruption, respond to the negative effects, and remain aware of changes in customer demand. Respondents were asked to rate the degree to which they agreed with the statements listed in the questionnaire (1 represented disagree and 5 represented strongly agree). The higher the rating, the higher the level of organizational resilience of the firm. 

#### 4.2.3. Servitization

This study measured servitization based on the survey items of Abou-foul et al. [65]. Servitization was measured based on six items, including the alignment of organizational structure with strategy, servitized offering, top management team support, and employee training. Respondents were asked to rate the degree to which they agreed with the statements listed in the questionnaire (1 represented disagree and 5 represented strongly agree). The higher the rating, the higher the level of servitization of the firm.

#### 4.2.4. Digitalization

The measurement of digitalization was based on Abou-foul et al. [65]. Specifically, this study measured digitalization of manufacturing firms using eight items, including the use of digital technologies in understanding customer needs, marketing and selling products and services through digital channels, using digital technologies in operational processes, using analytical tools for operational decision making, and launching new business models based on digital technologies. Respondents were asked to rate the degree to which they agreed with the statements listed in the questionnaire (1 represented disagree and 5 represented strongly agree). The higher the rating, the higher the level of digitalization of the firm. To mitigate concern regarding common method bias, we collected the patent data of 81 firms in our sample and compared the number of patents with our measure. The result shows that our measurement of digitalization was positively related to the number of patents of the company. 

#### 4.2.5. Environmental Dynamism

Similar to Chen et al. [55], this study measured environmental dynamism based on the rate of customers’ product/service needs change, suppliers’ skills/capabilities change, competitors’ products/services change, and a firm’s products/services change. Respondents were asked to rate the degree of change (volatility) in their business unit’s competitive environment relative to change in other industries (where 1 = very stable, 3 = about average for all industries, and 5 = very volatile). 

#### 4.2.6. Control Variables

This study controlled for several factors that might affect the results. First, this study controlled for firm size because larger firms tend to have more slack resources that can mitigate the impact of adversities [53]. Firm size was measured by coding the employee range based on the number of employees of the firm. The coding scheme is presented in Table 3. For this measure, larger numbers indicate larger firms. Second, this study controlled for firm age because old firms tend to be more experienced in dealing with different types of environmental changes. Firm age was measured by the natural logarithm of the number of years since the firm was established. Third, this study controlled for the competitive intensity. This was mainly because firms operating in a highly competitive environment might respond to adversities differently as compared with firms in less competitive environments. Competition intensity was measured based on Porter’s Five-Forces model [66], including competitive rivalry within the industry, market-entry, substitution threat, bargaining power of suppliers, and bargaining power of customers. Respondents were asked to indicate their perception of competitive pressure from the above five aspects based on five-point Likert scales, and then the average score was calculated to measure competitive intensity. Fourth, this study controlled for the adaptive capacity of the firm because adaptive capacity represents the firm’s dynamic capability, which enhances a firm’s ability to cope with dynamic environments [67]. Adaptive capacity was measured based on the scales used by Zhou and Li [68]. Finally, this study controlled the innovative capability of the firm, mainly because innovative firms tend to have more novel ideas to deal with environmental changes. Innovative capability was measured based on the scale used by Lin et al. [69]. 

The detailed measurement items are presented in the Appendix. The descriptive statistics of the variables are presented in Table 4. The correlation matrix of all variables is presented in Table 5. 

### 4.3. Reliability and Validity

We assessed the reliability of the study based on Cronbach’s alpha coefficients (presented in the Appendix A). The results indicated that all Cronbach’s alpha coefficients and composite reliability coefficients were larger than 0.7, indicating that the measurement items had good reliability. 

To measure the convergent validity, we constructed a confirmatory factor analysis (CFA) model with all variables included. The goodness-of-fit indices were: CMIN/df = 1.649, RMR = 0.090, IFI = 0.892, CFI = 0.890, and RMSEA = 0.063. Overall, the indices indicated that the data had a good fit with the measurement model. In addition, except for environmental dynamism, the average variance extracted (AVE) values of all constructs are larger than 0.5. The AVE of environmental dynamism was marginally smaller than 0.5 (0.494). However, the factor loadings of all items were higher than 0.5 and were significant at 0.05. The results indicated that convergent validity was not a serious concern.

We compared the square root of the AVE of each variable and its correlation with other variables to assess the discriminant validity of the measurements. The square roots of AVE are presented at the diagonal of the correlation matrix. The results indicated that the measurement items had good discriminant validity. 

### 4.4. Multicollinearity

The correlation matrix showed that the variables were correlated at a moderate level, indicating multicollinearity was not a major concern for this study. In addition, we mean centered variables before creating the interaction terms to mitigate multicollinearity between the independent variables and interaction terms. In all regression models, we used the variance inflation factor (VIF) coefficients to detect potential multicollinearity issues. 

### 4.5. Common Method Bias

Our results were prone to common method bias because the data on independent variables and dependent variables were collected from the same respondents. In this study, we followed the protocols suggested by Podsakoff et al. [70] to minimize common method bias. First, in the data collection stage, we ensured the anonymity of the respondents to motivate them to provide accurate and actual information about their company. Second, we conducted Harmon’s single factor test to detect common method variance; we conducted an exploratory factor analysis based on one factor and the results indicated that the first factor only explained 12.863% of the total variance, suggesting that common method variance did not explain all of the variances. Then, we conducted a confirmatory factor analysis based on a second-order common method model with one common method factor to explain all the variables. The results indicated that the common method model had a poor fit with the data. We also collected objective firm growth data of 113 firms and patent data of 81 firms. The results indicated that the subjective measure of firm growth was significantly related to the objective measure of firm growth. Furthermore, our measurement of digitalization was positively related to the number of patents of the firm. Overall, the results indicated that common method bias was not a major concern for this study. 

## 5. Results

### 5.1. The Impact of Service BMI and Digitalization on Organizational Resilience 

The regression results based on organizational resilience as the dependent variable are presented in Table 6. Model 1 includes the control variables; Models 2 and 3 include the independent variables. In Models 4 and 5, the interaction terms are included separately. Model 6 presents the regression results of the full model. The VIF coefficients in all models are smaller than five, indicating multicollinearity is not a concern in all regression models. Regarding control variables, the results indicate that competitive intensity is positively related to organizational resilience (β = 0.124, *p* < 0.1). In addition, firms with a strong innovation capability tend to have high levels of organizational resilience (β = 0.085, *p* < 0.05). Regarding the main effect, the results indicate that both servitization (β = 0.142, *p* < 0.01) and digitalization (β = 0.165, *p* < 0.01) are positively related to organizational resilience in all models. Therefore, both Hypothesis 1 and Hypothesis 2 are strongly supported.

### 5.2. Moderating Effects of Environmental Dynamism

As presented in Model 4–6 in Table 6, the interaction term of environmental dynamism and servitization is insignificant (β = 0.056, *p* > 0.1). Therefore, Hypothesis 3 is not supported. However, the interaction term of environmental dynamism and digitalization is positive and significant (β = 0.097, *p* < 0.05). Consistent with Hypothesis 4, the results indicate that environmental dynamism positively moderates the relationship between digitalization and organizational resilience.

**Figure 2:**
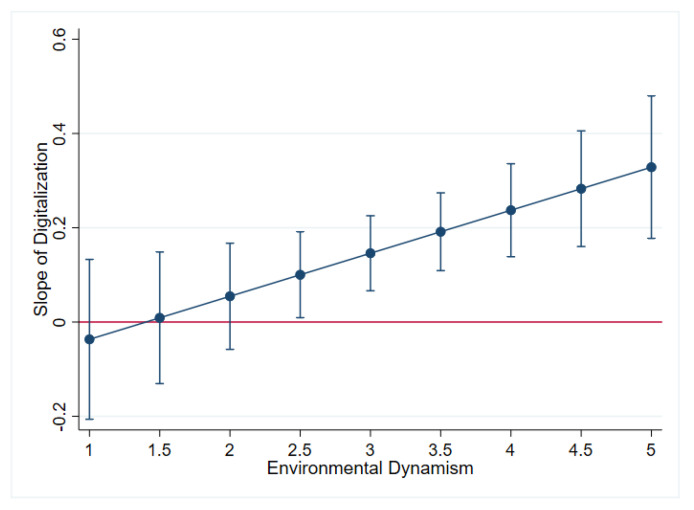
The slope of digitalization at different levels of environmental dynamism.

To further analyze the moderating effect of environmental dynamism, we conducted marginal effect analyses of the impact of digitalization on organizational resilience at different levels of environmental dynamism. The results are plotted in Figure 2. The marginal plot indicates that the impact of digitalization on organizational resilience is significant at high levels of environmental dynamism (larger than 2.5 in our data). This provides further support for Hypothesis 4. 

### 5.3. The Impact of Organizational Resilience on Firm Growth

Table 7 presents the regression results of models that take firm growth as the dependent variable. Model 1 includes the control variables. The results indicate that adaptive capacity (β = 0.589, *p* < 0.01) and innovation capability (β = 0.298, *p* < 0.05) are positively related to firm growth. In Model 2, servitization and digitalization are added. The results indicate that both servitization and digitalization are positively related to the firm growth of manufacturing firms. Model 3 presents the relationship between organizational resilience and firm growth. The results indicate that organizational resilience is positively related to firm growth (β = 1.200, *p* < 0.01). The results support Hypothesis 4. Model 4 presents the complete model with all independent variables included. The results indicate that the effect size of both servitization and digitalization reduces after adding organizational resilience. The results, together with the results presented in Table 3, indicate that organizational resilience partially mediates the impact of servitization and digitalization on firm growth.

Following Zhao et al. [71], we analyzed the indirect effects based on the bootstrap approach [72]. For servitization, the mean indirect effect from the bootstrap analysis is positive and significant (a × b = 0.073), with a 95% confidence interval excluding zero (0.023 to 0.151). The direct effect of servitization on firm growth is also positive and significant (c = 0.381). The total effect of servitization on firm growth is c’ = 0.455. It indicates that 16.04% of the effect of servitization on firm growth is mediated by organizational resilience. Since the indirect effect is positive, it is a complementary mediation. For digitalization, the mean indirect effect from the bootstrap analysis is positive and significant (a × b = 0.077), with a 95% confidence interval excluding zero (0.014 to 0.178). However, the direct effect of digitalization on firm growth is insignificant (c = 0.173), indicating it is an indirect-only mediation. 

## 6. Discussion

The importance of organizational resilience has been widely accepted, but research into the manner in which firms achieve resilience is lacking. In addition, the organizational resilience of healthcare firms has received scant attention. For medical device manufacturing firms, COVID-19 poses both threats and opportunities. The pandemic has caused widespread supply chain disruptions and reduced demand for some types of medical devices, for example, dental instruments and physical therapy apparatus, in the short term. At the same time, COVID-19 has raised awareness in the healthcare sector and will have a positive impact on the market prospects of healthcare devices in the long term. This study bridges the servitization, digitalization, and organizational resilience literature to examine whether servitization and digitalization can help manufacturing firms to enhance organizational resilience. According to a survey of 163 healthcare manufacturing firms conducted during the COVID-19 pandemic, we found that servitization and digitalization improve organizational resilience, which further improves firm growth. The impacts are more prominent for firms operating in dynamic environments.

### 6.1. Theoretical Implications

Our results have theoretical implications for firms seeking to deal with environmental adversities and build organizational resilience. First, this study suggests that organizational resilience is important for firms to maintain growth during the COVID-19 outbreak. This study addresses the need for further research on how firms cope with the disruptions caused by COVID-19. Previous studies have shown that firms with high organizational resilience are better able to mitigate the operational disruptions caused by the COVID-19 outbreak and to ensure the stability of operations. For example, Yu et al. [59] found that the resilience of the supply chain is positively related to the financial performance of manufacturing firms. Moreover, firms with high organizational resilience are more capable of capturing opportunities occurring during the global health crisis. Similarly, Zavala-Alcívar et al. [73] suggested that agility is a core element of supply chain resilience. In summary, this study offers an option for firms to cope with the challenges caused by the COVID-19 pandemic. Consistent with this stream of research, this study indicates that organizational resilience is also important for medical device manufacturing firms. We found that firms with higher levels of organizational resilience tend to experience higher levels of firm growth during the COVID-19 pandemic. Therefore, this study extends previous studies based on other industries. The medical device industry has specific characteristics that make it more prone to the disruptions caused by the COVID-19 outbreak. Intensive competition in the medical device industry makes firms compete aggressively and invest significant resources in R&D [74]. However, innovative technologies must be tested extensively before being introduced to the market [75]. Due to this extensive testing and the new product approval process, the pay-off period of these upfront investments in R&D is longer than that in other industries. This requires the firm to maintain a stable cash flow to maintain operations. Furthermore, there are high levels of regulation in the medical device industry [75]. Product quality risks that are harmful to consumers, i.e., patients, receive high penalties from the regulators. As a result, it is important to maintain a stable manufacturing process to minimize quality risks. In view of these two characteristics, maintaining high levels of organizational resilience, i.e., the stability and flexibility of operations, is important for firms to achieve sustainable competitive advantages. 

Second, the results indicate that servitization is positively related to organizational resilience. Consistent with previous studies that have emphasized the importance of diversification during changing environments [53], our study indicates that servitization, which refers to the diversification of business models, can help firms to mitigate the operational disruptions caused by the COVID-19 outbreak and achieve higher growth. On the basis of the diversification proposition, this study proposes a novel avenue for manufacturing firms to improve organizational resilience. In the healthcare industry, many medical device manufacturing firms have added services to their core products to create more value for customers, such as equipment maintenance and repair services, product-oriented training services, IT-supported remote services, and process-oriented training services [75]. As an extension to previous studies in servitization [20,37], this study indicates that in addition to financial outputs, servitization can also improve organizational resilience and help firms to cope better with environmental adversities. Therefore, our study expands the benefits of servitization for manufacturing firms and explores the mechanisms through which servitization can create long-term benefits for manufacturers. For medical device manufacturing firms, servitization is a new competitive strategy for dealing with intensive product competition. Adding services to the products can create more customer interaction and enhances customer stickiness, which enhances the competitive advantages of medical device manufacturing firms. 

Third, the results highlight the importance of digitalization for achieving high organizational resilience. Previous studies in organizational resilience have emphasized the importance of information-processing capability and proactive adaptation to anticipated environmental changes [76]. As an extension of these studies, this study empirically tested the impact of digitalization on the outcome of manufacturing firms during the COVID-19 outbreak. Widely adopted digital technologies have revolutionized the medical device industry. For example, the emergence of wearable devices such as smart watches enables firms to collect real-time data about customers and helps firms to provide more services to customers, such as health monitoring [77]. Similarly, social media platforms and big data analytics have helped firms to create user profiles and to design products that can capture diversified customer demand [78]. These technologies enable medical device firms to create more value for customers. In addition, through the adoption of digital technologies in the manufacturing processes, medical device firms can minimize quality risks and reduce manufacturing costs. For example, 3D printing technologies can improve the accuracy of manufacturing and have been used to produce dental and orthopedic instruments. The COVID-19 outbreak provides a different context to test the impact of digitalization. Our results indicate that firms equipped with more digital technologies are less likely to be affected by the operational disruptions caused by the COVID-19 outbreak. As a result, digitalization should be included as an important aspect of organizational resilience that enhances manufacturing firms’ ability to cope with adversities. Embracing digitalization can help medical device manufacturing firms to build sustainable competitive advantages. 

Finally, we examined the moderating effect of environmental dynamism on the impacts of digitalization and servitization on organizational resilience. We found that in a dynamic environment, digitalization has a stronger impact on organizational resilience, whereas the relationship between servitization and organizational resilience is not moderated by environmental dynamism. Our results echo previous studies that highlighted the need to improve information-processing capabilities to cope with uncertainties [27]. In this study, we suggest that digitalization can enhance the information processing capability and enables firms to build sustainable competitive advantages in the long term. In contrast, our results indicate that environmental dynamism does not affect the impact of servitization on organizational resilience. One potential explanation is that the outcome of servitization is mainly determined by the internal implementation and configuration of service strategy rather than the external environment. According to Zhang and Banerji [79], the major challenge for manufacturing firms is the reconfiguration of organizational structure, internal processes and capabilities, and supplier relationships. As a result, compared with digitalization, manufacturing firms encounter more challenges in the implementation and reconfiguration of servitization strategy. Therefore, the impact of digitalization is contingent upon environmental factors, whereas the impact of servitization is less likely to be affected by them. 

### 6.2. Practical Implications

This study offers two suggestions for medical device manufacturing firms to enhance organizational resilience and cope with adversities such as the COVID-19 outbreak. First, managers in medical device manufacturing firms should improve the resilience of the firm to cope with the recent COVID-19 pandemic. This study indicates that firms with higher levels of organizational resilience were able to achieve higher levels of firm growth during 2020. Building organizational resilience can enable firms to mitigate the negative impact caused by adversities and maintain the stable flow of operations. In addition, resilient firms are more capable of capturing opportunities during environmental adversities and build a more sustainable competitive advantage. Second, implementing servitization and digitalization can enhance the organizational resilience of manufacturing firms and help them adapt to internal and external disruptions. We suggest that manufacturing firms should implement servitization and digitalization to diversify their business models and enhance the information-processing capacities that help them to strengthen the resilience to environmental adversities. 

## 7. Conclusions

Mitigating the negative impact of adversities and identifying opportunities arising during crises has become a core issue for healthcare manufacturing firms. This study highlights the leading role of organizational resilience for maintaining firm growth by examining the impact of servitization and digitalization on organizational resilience. According to a survey study of 163 manufacturing firms conducted at the end of 2020, this study demonstrates that organizational resilience was positively related to firm growth during the COVID-19 pandemic. Furthermore, the results show that manufacturing firms can improve organizational resilience through servitization and digitalization. In a dynamic environment, digitalization has a stronger impact on organizational resilience. Thus, the results contribute more evidence about the importance of organizational resilience and broaden our understanding of servitization and digitalization. 

This study has several limitations that should be noted. First, our sample size was small and only covered a small range of industries. A small sample might limit the generalizability of the results. In addition, our results are prone to selection biases that we were unable to address due to the lack of valid instrumental variables. Although we controlled for several factors that might affect organizational resilience and firm growth, this stream of research would benefit from studies that rely on a larger sample to improve the external validity of the results. Second, our results were prone to common method bias. Although we conducted several tests to minimize common method concerns, the questions about the independent variables and dependent variables were answered by the same respondent. As a result, this study could not completely exclude common method bias. Future research should rely on data from diverse sources to obtain more solid results. The survey data should be combined with objective data collected from other sources to enhance the internal validity of the results. Specifically, Mènière et al. [80] provided a good method for measuring digitalization based on patent data. Future research can rely on this measure to obtain more robust results. Third, our results are prone to endogeneity concerns. Due to the cross-sectional design, we were unable to exclude alternative explanations and reverse causality, which reduced the internal validity of the results. A longitudinal design should be adopted to mitigate the potential issue of endogeneity of the results. Fourth, we treated organizational resilience as a single dimension construct. However, recent studies have suggested that organizational resilience is a multi-dimensional construct that contains elements such as anticipation, coping, and adaptation capabilities [24]. Future research should explore the nuances of resilience and examine how servitization and digitalization affect different dimensions of organizational resilience. Finally, in this study, we conceptualized organizational resilience in a general sense and did not consider the context in which organizational resilience might manifest. However, resilience is context dependent [22]. Firms may be more resilient to some types of adverse events but less prepared for others. To obtain a deeper understanding of the nuances of organizational resilience, future research should examine organizational resilience in other contexts. 

## Figures and Tables

**Table 1:** Product-based vs. service-based business models.

Dimensions	Product-Based Model	Service-Based Model
Customer interaction	Customer interaction through product selling.	More customer exchanges based on service delivery.
Predominant contractual relationship	Transactional (selling products)	Relational (selling integrated solutions through long-term relationships)
Focus of operation	Value co-creation focuses on assisting the customer in realizing the value potential embedded in products.	Co-creation of value in the customer-specific context.
Competitive positioning	Product as the core competitiveness.	Services are differentiated and part of a deliberate service strategy

**Table 2:** The product category distribution of the sample.

Product Categories	N	%
Medical diagnostic and monitoring instruments	50	30.67%
Physical therapy apparatus	27	16.56%
Dental instruments	36	22.09%
Disposable medical instruments	33	20.25%
Ophthalmic devices	13	7.98%
Others	4	2.45%
Total	163	100.00%

**Table 3:** Firm size distribution.

Code	Employee Range	Count	Percent	Cumulative
1	1–200	66	40.49%	40.49%
2	201–400	27	16.56%	57.06%
3	401–600	18	11.04%	68.10%
4	601–800	8	4.91%	73.01%
5	801–1000	7	4.29%	77.30%
6	>1000	37	22.70%	100.00%
	Total	163	100.00%	

**Table 4:** Descriptive statistics.

No	Variables	Mean	Std. Dev	Min	Median	Max	P25	P75
1	Firm growth	3.090	1.048	1.000	3.333	5.000	2.333	4.000
2	Org. Resil.	3.109	0.429	2.250	3.000	4.250	3.000	3.250
3	Servitization	2.685	0.975	1.000	2.833	4.833	1.833	3.500
4	Digitalization	3.112	0.963	1.000	3.167	5.000	2.333	3.833
5	Env. dynam.	3.183	0.824	1.000	3.250	5.000	2.500	3.750
6	Firm Size	2.840	2.012	1.000	2.000	6.000	1.000	5.000
7	Firm Age	3.397	0.769	1.386	3.401	5.193	2.944	3.951
8	Competition	3.315	0.502	1.800	3.200	4.600	3.000	3.600
9	Adaptive cap.	3.359	0.860	1.000	3.250	5.000	3.000	4.000
10	Inno. cap.	3.201	0.948	1.000	3.250	5.000	2.750	4.000

Note: N = 163. P25, the first quartile and P75, the third quartile. Org. Resil., organizational resilience; Env. Dynam., environmental dynamism; Adaptive Cap., adaptive capability; Inno. Cap., innovative capability.

**Table 5:** Correlation matrix.

No	Variables	1	2	3	4	5	6	7	8	9	10
1	Firm Growth	(0.82)									
2	Org. Resil.	0.394	(0.71)								
3	Servitization	0.526	0.332	(0.71)							
4	Digitalization	0.482	0.405	0.333	(0.74)						
5	Env. Dynam.	0.222	0.331	0.209	0.171	(0.70)					
6	Firm Size	0.132	0.069	0.236	0.263	−0.009	(n.a.)				
7	Firm Age	−0.083	−0.044	0.079	−0.032	−0.141	0.077	(n.a.)			
8	Competition	0.127	0.202	0.219	0.155	0.091	0.088	0.033	(n.a.)		
9	Adaptive cap.	0.445	0.251	0.283	0.614	0.268	0.123	−0.122	0.157	(0.81)	
10	Inno. cap.	0.331	0.294	0.225	0.491	0.285	0.104	−0.067	0.136	0.484	(0.81)

Note: N = 163. All coefficients larger than 0.15 are significant at 0.05. Parenthesis indicates square root AVE values of the individual constructs. Org. Resil., organizational resilience; Env. Dynam., environmental dynamism; Adaptive Cap., adaptive capability; Inno. Cap., innovative capability; n.a., not applicable (single items).

**Table 6:** Regression results (organizational resilience as DV).

Independent Variables	Dependent Variable: Organizational resilience					
	Model 1	Model 2	Model 3	Model 4	Model 5	Model 6
Firm size	0.009	−0.006	−0.005	−0.003	−0.006	−0.005
	(0.560)	(−0.396)	(−0.340)	(−0.172)	(−0.440)	(−0.388)
Firm age	−0.000	−0.016	0.002	−0.005	−0.002	−0.004
	(−0.008)	(−0.469)	(0.064)	(−0.135)	(−0.071)	(−0.111)
Competition	0.129 *	0.077	0.071	0.070	0.056	0.057
	(1.805)	(1.225)	(1.188)	(1.166)	(0.957)	(0.958)
Adaptive capacity	0.056	−0.047	−0.067*	−0.060	−0.063 *	−0.062
	(1.379)	(−1.151)	(−1.693)	(−1.456)	(−1.668)	(−1.589)
Innovation capability	0.086 **	0.020	0.002	0.004	0.024	0.023
	(2.359)	(0.609)	(0.065)	(0.113)	(0.666)	(0.645)
Servitization		0.142 ***	0.119 ***	0.121 ***	0.115 ***	0.116 ***
		(3.873)	(3.341)	(3.382)	(3.217)	(3.245)
Digitalization		0.167 ***	0.172 ***	0.163 ***	0.167 ***	0.165 ***
		(4.031)	(4.327)	(3.928)	(4.378)	(4.223)
Env. Dynam.			0.125 ***	0.121 ***	0.116 ***	0.116 ***
			(3.461)	(3.523)	(3.521)	(3.545)
Serv. × Env. Dynam.				0.051		0.012
				(1.504)		(0.337)
Digit × Env. Dynam.					0.097 ***	0.093 ***
					(3.311)	(2.972)
Category dummies	Included	Included	Included	Included	Included	Included
Constant	1.854 ***	1.708 ***	1.449 ***	1.475 ***	1.470 ***	1.475 ***
	(5.458)	(5.547)	(4.642)	(4.797)	(5.042)	(5.039)
N	163	163	163	163	163	163
R-squared	0.172	0.332	0.379	0.387	0.414	0.415
Adj. R-squared	0.117	0.278	0.325	0.329	0.359	0.355
F	7.419	7.253	8.432	8.401	9.335	8.746

Note: * *p* < 0.1, ** *p* < 0.05, *** *p* < 0.01. The t-statistics (based on robust standard errors) are in parentheses below regression coefficients. Env. Dynam., environmental dynamism; Serv., servitization; Digit, digitalization.

**Table 7:** Regression results (firm growth as DV).

Independent Variables	Dependent Variable: Firm Growth					
	Model 1	Model 2	Model 3	Model 4	Model 5	Model 6
Firm size	0.011	−0.019	0.003	−0.017	−0.014	−0.011
	(0.302)	(−0.556)	(0.085)	(−0.496)	(−0.386)	(−0.317)
Firm age	−0.050	−0.103	−0.065	−0.104	−0.117	−0.115
	(−0.516)	(−1.264)	(−0.731)	(−1.306)	(−1.431)	(−1.440)
Competition	0.060	−0.080	−0.030	−0.111	−0.078	−0.104
	(0.344)	(−0.515)	(−0.190)	(−0.757)	(−0.505)	(−0.703)
Adaptive capacity	0.374 ***	0.203 *	0.348 ***	0.233 **	0.218 *	0.247 **
	(3.308)	(1.842)	(3.228)	(2.107)	(1.975)	(2.256)
Innovation capability	0.179 **	0.060	0.129	0.059	0.056	0.045
	(2.084)	(0.738)	(1.582)	(0.731)	(0.661)	(0.540)
Env. Dynam.	0.135	0.051	0.019	−0.005	0.044	−0.009
	(1.515)	(0.600)	(0.210)	(−0.061)	(0.513)	(−0.107)
Servitization		0.435 ***		0.381 ***	0.439 ***	0.385 ***
		(4.764)		(4.021)	(4.892)	(4.127)
Digitalization		0.247 **		0.170	0.228 **	0.151
		(2.477)		(1.607)	(2.299)	(1.454)
Org. resilience			0.791 ***	0.447 **		0.464 **
			(4.606)	(2.287)		(2.345)
Serv. × Env. Dynam.					0.113	0.107
					(1.344)	(1.266)
Digit × Env. Dynam.					−0.033	−0.076
					(−0.406)	(−0.926)
Category dummies	Included	Included	Included	Included	Included	Included
Constant	1.239	1.114	0.018	0.466	1.166	0.481
	(1.371)	(1.481)	(0.022)	(0.604)	(1.513)	(0.603)
N	163	163	163	163	163	163
R-squared	0.290	0.438	0.370	0.459	0.444	0.465
Adj. R-squared	0.238	0.389	0.319	0.408	0.387	0.406
F	6.407	13.438	8.967	12.923	12.908	11.830

Note: * *p* < 0.1, ** *p* < 0.05, *** *p* < 0.01. The t-statistics (based on robust standard errors) are in parentheses below regression coefficients.

## Data Availability

The survey data used to support the findings of this study are available from the corresponding author upon request.

## Appendix A. Measurement Items

The measurement items and standardized factor loadings in the CFA model are presented in the table below.

**Table A1:** Measurement items.

**Variables and Items**	**Factor** **Loadings**
**Servitization** (Cronbach’s alpha = 0.873, CR = 0.870, AVE = 0.501)	
1. Our senior leaders are aligned around the strategic importance of servitization transformation.	0.627
2. Our senior leaders are actively promoting a vision of the future that involves servitized offerings.	0.798
3. We regularly review with the top team our progress on servitization transformation.	0.715
4. Our employees understand the benefits of servitization change.	0.744
6. Our firm has taken over some of our customers’ business processes.	0.691
7. Our firm has taken over the operational functions of our products in customers’ businesses.	0.675
**Digitalization** (Cronbach’s alpha = 0.894, CR = 0.907, AVE = 0.554)	
1. We are using digital technologies (such as analytics, social media, mobile, and embedded devices) to understand our customers better.	0.645
2. We market and sell our products and services through digital channels.	0.827
3. We use digital channels to provide customer service.	0.878
4. Technology is allowing us to link customer-facing and operational processes in new ways.	0.822
5. Our core processes are automated.	0.661
6. We use analytics to make better operational decisions.	0.588
7. We use digital technologies to increase the performance of our existing products and services.	0.645
8. We have launched new business models based on digital technologies.	0.827
**Organizational resilience** (Cronbach’s alpha = 0.713, CR = 0.802, AVE = 0.504)	
1. We can cope with changes in our business brought on by COVID-19.	0.768
2. We can easily adapt our business operations to the disruption caused by COVID-19.	0.729
3. We can provide a quick response to the negative effects of a COVID-19 disruption on our business.	0.665
4. We always remain aware of changes in customer demand.	0.675
**Environmental dynamism** (Cronbach’s alpha = 0.793, CR = 0.796, AVE = 0.494)	
1. The rate at which your customers’ product/service needs change.	0.744
2. The rate at which your suppliers’ skills/capabilities change.	0.745
3. The rate at which your competitors’ products/services change.	0.649
4. The rate at which your firm’s products/services change.	0.670
**Firm growth** (Cronbach’s alpha = 0.853, CR = 0.861, AVE = 0.673)	
1. Sales growth compared to 2019	0.857
2. Market expansion (percentage of growth coming from new markets entered compared to 2019)	0.788
3. Market share growth compared to 2019	0.815
**Innovation capability** (Cronbach’s alpha = 0.880, CR = 0.885, AVE = 0.659)	
1. Our company launches new products frequently.	0.738
2. Our company extends the number of product lines.	0.761
3. Our company engages in NPD to obtain patents.	0.882
4. Our company launches customized products according to market demands.	0.856
**Adaptive capacity** (Cronbach’s alpha = 0.882, CR = 0.884, AVE = 0.657)	
1. We can react properly to changes in the market.	0.788
2. Our existing competency can withstand changes in the industry.	0.812
3. Our existing competency can withstand the challenges caused by environmental changes.	0.857
4. Our existing competency can withstand the challenges brought about by the e-commerce trend.	0.782

## References

[1] El Baz J., Ruel S. (2021). Can supply chain risk management practices mitigate the disruption impacts on supply chains’ resilience and robustness? Evidence from an empirical survey in a COVID-19 outbreak era. Int. J. Prod. Econ..

[2] Kim H.K., Lee C.W. (2021). Relationships among healthcare digitalization, social capital, and supply chain performance in the healthcare manufacturing industry. Int. J. Environ. Res. Public Health.

[3] Financial-Times Prospering in the Pandemic: The Top 100 Companies. https://www.ft.com/content/844ed28c-8074-4856-bde0-20f3bf4cd8f0.

[4] Hillmann J., Guenther E. (2020). Organizational resilience: A valuable construct for management research?. Int. J. Manag. Rev..

[5] Madrigano J., Chandra A., Costigan T., Acosta J.D. (2017). Beyond disaster preparedness: Building a resilience-oriented workforce for the future. Int. J. Environ. Res. Public Health.

[6] Williams T.A., Gruber D.A., Sutcliffe K.M., Shepherd D.A., Zhao E.Y. (2017). Organizational response to adversity: Fusing crisis management and resilience research streams. Acad. Manag. Ann..

[7] Fernandes N. (2020). Economic effects of coronavirus outbreak (COVID-19) on the world economy. Available SSRN 3557504.

[8] Visnjic I., Wiengarten F., Neely A. (2016). Only the brave: Product innovation, service business model innovation, and their impact on performance. J. Prod. Innov. Manag..

[9] Parida V., Sjödin D., Reim W. (2019). Reviewing Literature on Digitalization, Business Model Innovation, and Sustainable Industry: Past Achievements and Future Promises. Sustainability.

[10] Kohtamäki M., Parida V., Patel P.C., Gebauer H. (2020). The relationship between digitalization and servitization: The role of servitization in capturing the financial potential of digitalization. Technol. Forecast. Soc. Chang..

[11] Miceli A., Hagen B., Riccardi M.P., Sotti F., Settembre-Blundo D. (2021). Thriving, Not Just Surviving in Changing Times: How Sustainability, Agility and Digitalization Intertwine with Organizational Resilience. Sustainability.

[12] Pfeffer J., Salancik G.R. (2003). The External Control of Organizations: A Resource Dependence Perspective.

[13] Vendrell-Herrero F., Bustinza O.F., Parry G., Georgantzis N. (2017). Servitization, digitization and supply chain interdependency. Ind. Mark. Manag..

[14] Rapaccini M., Saccani N., Kowalkowski C., Paiola M., Adrodegari F. (2020). Navigating disruptive crises through service-led growth: The impact of COVID-19 on Italian manufacturing firms. Ind. Mark. Manag..

[15] Joseph J., Gaba V. (2020). Organizational structure, information processing, and decision-making: A retrospective and road map for research. Acad. Manag. Ann..

[16] Liu T.-H., Hung S.-C., Chu Y.-Y. (2007). Environmental jolts, entrepreneurial actions and value creation: A case study of Trend Micro. Technol. Forecast. Soc. Chang..

[17] DesJardine M., Bansal P., Yang Y. (2019). Bouncing back: Building resilience through social and environmental practices in the context of the 2008 global financial crisis. J. Manag..

[18] Parker H., Ameen K. (2018). The role of resilience capabilities in shaping how firms respond to disruptions. J. Bus. Res..

[19] Fan H., Li G., Sun H., Cheng T. (2017). An information processing perspective on supply chain risk management: Antecedents, mechanism, and consequences. Int. J. Prod. Econ..

[20] Wang W., Lai K.-H., Shou Y. (2018). The impact of servitization on firm performance: A meta-analysis. Int. J. Oper. Prod. Manag..

[21] Kossek E.E., Perrigino M.B. (2016). Resilience: A review using a grounded integrated occupational approach. Acad. Manag. Ann..

[22] Linnenluecke M.K. (2017). Resilience in business and management research: A review of influential publications and a research agenda. Int. J. Manag. Rev..

[23] Richtnér A., Löfsten H. (2014). Managing in turbulence: How the capacity for resilience influences creativity. RD Manag..

[24] Duchek S., Raetze S., Scheuch I. (2020). The role of diversity in organizational resilience: A theoretical framework. Bus. Res..

[25] Jüttner U., Maklan S. (2011). Supply chain resilience in the global financial crisis: An empirical study. Supply Chain Manag..

[26] Srinivasan R., Swink M. (2018). An investigation of visibility and flexibility as complements to supply chain analytics: An organizational information processing theory perspective. Prod. Oper. Manag..

[27] Galbraith J.R. (1974). Organization design: An information processing view. Interfaces.

[28] Wong C.W., Lirn T.-C., Yang C.-C., Shang K.-C. (2020). Supply chain and external conditions under which supply chain resilience pays: An organizational information processing theorization. Int. J. Prod. Econ..

[29] Dubey R., Gunasekaran A., Childe S.J., Fosso Wamba S., Roubaud D., Foropon C. (2021). Empirical investigation of data analytics capability and organizational flexibility as complements to supply chain resilience. Int. J. Prod. Res..

[30] Yu W., Zhao G., Liu Q., Song Y. (2021). Role of big data analytics capability in developing integrated hospital supply chains and operational flexibility: An organizational information processing theory perspective. Technol. Forecast. Soc. Chang..

[31] Foss N.J., Saebi T. (2017). Fifteen years of research on business model innovation: How far have we come, and where should we go?. J. Manag..

[32] Zott C., Amit R., Massa L. (2011). The business model: Recent developments and future research. J. Manag..

[33] Baines T., Bigdeli A.Z., Sousa R., Schroeder A. (2020). Framing the servitization transformation process: A model to understand and facilitate the servitization journey. Int. J. Prod. Econ..

[34] Harris W.L., Wonglimpiyarat J. (2020). Strategic foresight of Xerox servitization: Look back and look forward. Foresight.

[35] Neely A. (2008). Exploring the financial consequences of the servitization of manufacturing. Oper. Manag. Res..

[36] Fang E., Palmatier R.W., Steenkamp J.-B.E. (2008). Effect of service transition strategies on firm value. J. Mark..

[37] Visnjic I.K., Van Looy B. (2013). Servitization: Disentangling the impact of service business model innovation on manufacturing firm performance. J. Oper. Manag..

[38] Barney J. (1991). Firm resources and sustained competitive advantage. J. Manag..

[39] Eggert A., Hogreve J., Ulaga W., Muenkhoff E. (2014). Revenue and profit implications of industrial service strategies. J. Serv. Res..

[40] Gebauer H., Paiola M., Saccani N., Rapaccini M. (2021). Digital servitization: Crossing the perspectives of digitization and servitization. Ind. Mark. Manag..

[41] Hallstedt S.I., Isaksson O., Öhrwall Rönnbäck A. (2020). The Need for New Product Development Capabilities from Digitalization, Sustainability, and Servitization Trends. Sustainability.

[42] Isensee C., Teuteberg F., Griese K.-M., Topi C. (2020). The relationship between organizational culture, sustainability, and digitalization in SMEs: A systematic review. J. Clean. Prod..

[43] Paiola M., Gebauer H. (2020). Internet of things technologies, digital servitization and business model innovation in BtoB manufacturing firms. Ind. Mark. Manag..

[44] Ardolino M., Rapaccini M., Saccani N., Gaiardelli P., Crespi G., Ruggeri C. (2018). The role of digital technologies for the service transformation of industrial companies. Int. J. Prod. Res..

[45] Amit R., Zott C. (2012). Creating value through business model innovation. MIT Sloan Manag. Rev..

[46] Sousa R., da Silveira G.J. (2019). The relationship between servitization and product customization strategies. Int. J. Oper. Prod. Manag..

[47] Kastalli I.V., Van Looy B., Neely A. (2013). Steering manufacturing firms towards service business model innovation. Calif. Manag. Rev..

[48] Sousa R., da Silveira G.J. (2017). Capability antecedents and performance outcomes of servitization: Differences between basic and advanced services. Int. J. Oper. Prod. Manag..

[49] Moeller S. (2008). Customer integration—a key to an implementation perspective of service provision. J. Serv. Res..

[50] Saccani N., Visintin F., Rapaccini M. (2014). Investigating the linkages between service types and supplier relationships in servitized environments. Int. J. Prod. Econ..

[51] Zhang M., Huo B. (2013). The impact of dependence and trust on supply chain integration. Int. J. Phys. Distrib. Logist. Manag..

[52] Ambulkar S., Blackhurst J., Grawe S. (2015). Firm’s resilience to supply chain disruptions: Scale development and empirical examination. J. Oper. Manag..

[53] Hendricks K.B., Singhal V.R., Zhang R. (2009). The effect of operational slack, diversification, and vertical relatedness on the stock market reaction to supply chain disruptions. J. Oper. Manag..

[54] Bragazzi N.L. (2020). Digital Technologies-Enabled Smart Manufacturing and Industry 4.0 in the Post-COVID-19 Era: Lessons Learnt from a Pandemic. Int. J. Environ. Res. Public Health.

[55] Chen D.Q., Preston D.S., Swink M. (2015). How the use of big data analytics affects value creation in supply chain management. J. Manag. Inf. Syst..

[56] Bundy J., Pfarrer M.D., Short C.E., Coombs W.T. (2017). Crises and crisis management: Integration, interpretation, and research development. J. Manag..

[57] Ataseven C., Nair A. (2017). Assessment of supply chain integration and performance relationships: A meta-analytic investigation of the literature. Int. J. Prod. Econ..

[58] Richard O.C., Wu J., Markoczy L.A., Chung Y. (2019). Top management team demographic-faultline strength and strategic change: What role does environmental dynamism play?. Strat. Manag. J..

[59] Yu W., Jacobs M.A., Chavez R., Yang J. (2019). Dynamism, disruption orientation, and resilience in the supply chain and the impacts on financial performance: A dynamic capabilities perspective. Int. J. Prod. Econ..

[60] Alkahtani M., Omair M., Khalid Q.S., Hussain G., Ahmad I., Pruncu C. (2021). A covid-19 supply chain management strategy based on variable production under uncertain environment conditions. Int. J. Environ. Res. Public Health.

[61] Dubey R., Gunasekaran A., Childe S.J., Bryde D.J., Giannakis M., Foropon C., Roubaud D., Hazen B.T. (2020). Big data analytics and artificial intelligence pathway to operational performance under the effects of entrepreneurial orientation and environmental dynamism: A study of manufacturing organisations. Int. J. Prod. Econ..

[62] Ortiz-de-Mandojana N., Bansal P. (2016). The long-term benefits of organizational resilience through sustainable business practices. Strat. Manag. J..

[63] Dyer J.H., Singh H. (1998). The relational view: Cooperative strategy and sources of interorganizational competitive advantage. Acad. Manag. Rev..

[64] Ketokivi M. (2019). Avoiding bias and fallacy in survey research: A behavioral multilevel approach. J. Oper. Manag..

[65] Abou-foul M., Ruiz-Alba J.L., Soares A. (2020). The impact of digitalization and servitization on the financial performance of a firm: An empirical analysis. Prod. Plan. Control.

[66] Porter M.E. (1980). Competitive Strategy: Techniques for Analyzing Industry and Competitors.

[67] Wang C.L., Ahmed P.K. (2007). Dynamic capabilities: A review and research agenda. Int. J. Manag. Rev..

[68] Zhou K.Z., Li C.B. (2010). How strategic orientations influence the building of dynamic capability in emerging economies. J. Bus. Res..

[69] Lin R.J., Chen R.H., Chiu K.K.S. (2010). Customer relationship management and innovation capability: An empirical study. Ind. Manag. Data Syst..

[70] Podsakoff P.M., MacKenzie S.B., Lee J.-Y., Podsakoff N.P. (2003). Common method biases in behavioral research: A critical review of the literature and recommended remedies. J. Appl. Psychol..

[71] Zhao X., Lynch J.G., Chen Q. (2010). Reconsidering Baron and Kenny: Myths and truths about mediation analysis. J. Consum. Res..

[72] Preacher K.J., Hayes A.F. (2008). Asymptotic and resampling strategies for assessing and comparing indirect effects in multiple mediator models. Behav. Res. Methods.

[73] Zavala-Alcívar A., Verdecho M.-J., Alfaro-Saíz J.-J. (2020). A conceptual framework to manage resilience and increase sustainability in the supply chain. Sustainability.

[74] Ball G.P., Shah R., Wowak K.D. (2018). Product competition, managerial discretion, and manufacturing recalls in the US pharmaceutical industry. J. Oper. Manag..

[75] Schröter M., Lay G. (2014). Manufacturers of medical technology: Servitization in regulated markets. Servitization in Industry.

[76] Chowdhury M.M.H., Quaddus M. (2017). Supply chain resilience: Conceptualization and scale development using dynamic capability theory. Int. J. Prod. Econ..

[77] Marakhimov A., Joo J. (2017). Consumer adaptation and infusion of wearable devices for healthcare. Comput. Hum. Behav..

[78] Han Z.-H., Chen X.-S., Zeng X.-M., Zhu Y., Yin M.-Y. (2019). Detecting proxy user based on communication behavior portrait. Comput. J..

[79] Zhang W., Banerji S. (2017). Challenges of servitization: A systematic literature review. Ind. Mark. Manag..

[80] Mènière Y., Rudyk I., Valdes J. (2017). Patents and the Fourth Industrial Revolution: The Inventions Behind Digital Transformation.

